# Gaps in health security related to wildlife and environment affecting pandemic prevention and preparedness, 2007–2020

**DOI:** 10.2471/BLT.20.272690

**Published:** 2021-03-02

**Authors:** Catherine Machalaba, Marcela Uhart, Marie-Pierre Ryser-Degiorgis, William B Karesh

**Affiliations:** a EcoHealth Alliance, 520 Eighth Avenue, Suite 1200, New York, NY 10018, United States of America (USA).; b One Health Institute, School of Veterinary Medicine, University of California, Davis, USA.; c Centre for Fish and Wildlife Health, Department of Infectious Diseases and Pathobiology, University of Bern, Bern, Switzerland.

## Abstract

**Objective:**

To describe and quantify the extent of wildlife and environment sector inclusion in country evaluation and prioritization tools for health security, and to provide practical recommendations for global and national action to improve pandemic prevention and preparedness.

**Methods:**

To assess coverage of wildlife and other environmental aspects, we reviewed major health security reports (including World Organisation for Animal Health Performance of Veterinary Services reports, and World Health Organization Joint External Evaluations and follow-on National Action Plans for Health Security) published by 107 countries and territories. We extracted information on stated coverage gaps, wildlife surveillance systems and priority diseases. We also searched National Biodiversity Strategies and Action Plans published by 125 countries to assess whether disease surveillance or prevention activities were included.

**Findings:**

We noted that the occurrence frequency of keywords indicative of wildlife, environment, biodiversity and climate factors varied with type of report and between countries. We found that more than half (57.9%, 62/107) of the reporting countries did not provide any evidence of a functional wildlife health surveillance programme. Most countries (83.2%, 89/107) indicated specific gaps in operations, coordination, scope or capacity. Only eight of the 125 countries (6.4%) publishing a National Biodiversity Strategy and Action Plan reported tangible activities related to wildlife health or zoonotic disease.

**Conclusion:**

Overall, despite their importance for pandemic prevention, wildlife and environmental considerations are neglected in health security priorities and plans. Strengthening wildlife health capacity and operations should be emphasized in One Health efforts to monitor and mitigate known and novel disease risks.

## Résumé

**Objectif:**

Décrire et déterminer dans quelle mesure la faune sauvage et l'environnement sont intégrés aux outils d'évaluation et de définition des priorités des pays dans le domaine de la sécurité sanitaire ; formuler des recommandations pratiques pour entreprendre des actions nationales et internationales visant à mieux prévenir les pandémies et à assurer une meilleure préparation.

**Méthodes:**

Afin d'évaluer la prise en considération de la faune sauvage et d'autres aspects environnementaux, nous avons examiné les principaux rapports de sécurité sanitaire (notamment les rapports de performance des services vétérinaires de l'Organisation Mondiale de la Santé animale, les évaluations externes conjointes de l'Organisation mondiale de la Santé et le suivi des Plans d'action nationaux pour la sécurité sanitaire) publiés par 107 pays et territoires. Nous y avons prélevé des informations sur le manque de couverture affiché, les systèmes de surveillance de la faune sauvage et les maladies prioritaires. Nous avons également étudié les stratégies nationales et plans d'action pour la biodiversité parus dans 125 pays en vue d'y déceler d'éventuelles activités de prévention et de surveillance des maladies.

**Résultats:**

Nous avons constaté que la fréquence d'apparition de mots clés faisant référence à la faune sauvage, l'environnement, la biodiversité et les facteurs climatiques variait en fonction du type de rapport et du pays. Plus de la moitié (57,9%, 62/107) des pays d'où provenaient les rapports ne mentionnaient aucune indication de l'existence d'un programme contrôlant le bon fonctionnement de la faune sauvage. La plupart des pays (83,2%, 89/107) ont signalé des lacunes spécifiques au niveau des opérations, de la coordination, du cadre ou des capacités. Seuls 8 pays sur 125 (6,4%) ayant publié une stratégie nationale et un plan d'action pour la biodiversité ont fait état d'activités tangibles liées à la santé des espèces sauvages ou aux zoonoses.

**Conclusion:**

Globalement, malgré leur importance en matière de prévention des pandémies, les considérations relatives à la faune sauvage et à l'environnement font rarement partie des priorités et plans de sécurité sanitaire. Le renforcement des capacités et opérations préservant la santé des espèces sauvages devrait faire partie intégrante des efforts de l'initiative «One Health» destinée à surveiller et limiter les risques de maladies, qu'ils soient connus ou émergents.

## Resumen

**Objetivo:**

Describir y determinar el grado de integración de la fauna silvestre y el medio ambiente en las herramientas de evaluación y priorización de la seguridad sanitaria de los países; ofrecer recomendaciones prácticas para las acciones nacionales e internacionales orientadas a mejorar la prevención de pandemias y garantizar una mejor preparación.

**Métodos:**

Para evaluar la consideración de la fauna silvestre y otros aspectos medioambientales, hemos revisado los principales informes de seguridad sanitaria (incluidos los informes de rendimiento del Proceso de Prestaciones de los Servicios Veterinarios de la Organización Mundial de Sanidad Animal, las herramientas de evaluación externa conjunta de la Organización Mundial de la Salud y el seguimiento de los planes de acción nacionales en pro de la seguridad sanitaria) publicados por 107 países y territorios. Se extrajo información sobre las deficiencias de cobertura mencionadas, los sistemas de vigilancia en la fauna silvestre y las enfermedades prioritarias. También revisamos las estrategias y los planes de acción nacionales sobre biodiversidad de 125 países para identificar posibles actividades de prevención y control de enfermedades.

**Resultados:**

Comprobamos que la frecuencia de aparición de las palabras clave referidas a la fauna silvestre el medio ambiente, la biodiversidad y los factores climáticos fue variable según el tipo de informe y el país. Más de la mitad (57,9%, 62/107) de los países de los que proceden los informes no presentan evidencia de la existencia de un programa de vigilancia en fauna silvestre. La mayoría de los países (83,2%, 89/107) señalaron deficiencias específicas en las operaciones, la coordinación, el marco o la capacidad. Solo 8 de los 125 países (6,4%) que habían publicado una estrategia y un plan de acción nacionales para la biodiversidad informaron de actividades tangibles relacionadas con la salud de la fauna silvestre o las zoonosis.

**Conclusión:**

En general, a pesar de su importancia en la prevención de pandemias, las consideraciones sobre la fauna silvestre y el medio ambiente rara vez se incluyen en las prioridades y planes de seguridad sanitaria. El refuerzo de las capacidades y funcionamiento en materia de salud de la fauna silvestre debe ser integrada a los esfuerzos Una Salud para controlar y limitar el riesgo de enfermedades existentes y emergentes.

## ملخص

الغرض وصف وإحصاء مدى مشاركة قطاع الحياة البرية والبيئة في أدوات التقييم وتحديد الأولويات في البلد للأمن الصحي، وتقديم توصيات عملية للعمل العالمي والوطني لتحسين الوقاية من الوباء والاستعداد له.

الطريقة لتقييم تغطية الحياة البرية والجوانب البيئية الأخرى، راجعنا التقارير الرئيسية عن الأمن الصحي العالمي (بما في ذلك التقارير عن أداء الخدمات البيطرية الصادرة من المنظمة العالمية لصحة الحيوان، والتقييمات الخارجية المشتركة الصادرة عن منظمة الصحة العالمية ومتابعة خطط العمل الوطنية للأمن الصحي) والمنشورة من جانب 107 دول ومناطق. استقينا معلومات عن الفجوات المذكورة في التغطية ونظم مراقبة الحياة البرية والأمراض التي لها الأولوية. كما أننا بحثنا في الاستراتيجيات وخطط العمل الوطنية للتنوع الحيوي المنشورة من جانب 125 دولة لتقييم ما إذا كانت نشاطات مراقبة الأمراض أو الوقاية منها مدرجة.

النتائج لاحظنا أن معدل تكرار الكلمات الأساسية التي تشير إلى الحياة البرية والبيئة والتنوع الحيوي والعوامل المناخية اختلف على حسب نوع التقرير وفي ما بين الدول. وجدنا أن أكثر من نصف الدول التي قدمت تقريرًا (57,9%، 62/107) لم تقدم أي دليل على وجود برنامج فعال لمراقبة صحة الحياة البرية. أشارت معظم الدول (83,2%، 89/107) إلى فجوات محددة في العمليات أو التنسيق أو النطاق أو السعة. أبلغت 8 دول فقط من 125 دولة (6,4%) نشرت استراتيجية وخطة عمل وطنيتين للتنوع الحيوي ترتبطان بصحة الحياة البرية أو المرض حيواني المصدر. ****

الاستنتاج تتعرض الاعتبارات الخاصة بالحياة البرية والاعتبارات البيئية للتجاهل بشكل عام في الأولويات والخطط الخاصة بالأمن الصحي على الرغم من أهميتها للوقاية من الجائحة. ينبغي التأكيد على تعزيز القدرات والعمليات الخاصة بصحة الحياة البرية في جهود مبادرة الصحة الواحدة لمراقبة المخاطر الناتجة عن الأمراض المعروفة والجديدة والحد منها.

## 摘要

**目的:**

旨在描述和量化国家评估卫生安全及为其选择优选工具时对野生动物和环境因素的重视程度，并为全球和国家采取行动以加强流行病预防和防范提供切实可行的建议。

**方法:**

我们回顾了 107 个国家和地区发表的主要卫生安全报告（包括世界动物卫生组织兽医服务体系效能报告以及世界卫生组织联合外部评估和后续的《国家卫生安全行动计划》），对野生动物和其他环境因素的覆盖面进行了评估。我们提取了与上述覆盖面差距、野生动物监测系统和重点疾病相关的信息。我们还搜索了 125 个国家/地区发布的国家生物多样性战略和行动计划，以评估是否包括疾病监测或预防行为。

**结果:**

我们注意到，野生动物、环境、生物多样性和气候因素相关关键字的出现频率因报告类型和国家/地区而异。我们发现，超过一半 (57.9％, 62/107) 的报告国家未提供任何有效的野生动物卫生监测计划证据。研究表明，大多数国家 (83.2％, 89/107) 特别是在行动、协作、范围或能力方面存在差距。在发布国家生物多样性战略和行动计划的 125 个国家中，仅 8 个国家 (6.4％) 就野生动物卫生或人畜共患病报告了相关具体行动。

**结论:**

总之，尽管野生动物和环境因素在预防流行病方面具有重要意义，但在卫生安全重点和计划中却未得到重视。在“同一个健康”行动中，应着重于强化野生动物健康防疫能力和相关举措，以监测并减轻已知和新型疾病风险。

## Резюме

**Цель:**

Описать и количественно оценить степень включения сектора дикой природы и окружающей среды в инструменты оценки и определения приоритетов в области безопасности здравоохранения стран, а также предоставить практические рекомендации по глобальным и национальным действиям по улучшению профилактики пандемий и обеспечению готовности к ним.

**Методы:**

Чтобы оценить освещение событий относительно дикой природы и других аспектов окружающей среды, авторы изучили основные отчеты о безопасности здравоохранения (включая отчеты Всемирной организации по охране здоровья животных, а также совместные внешние оценки Всемирной организации здравоохранения и последующие национальные планы действий по обеспечению безопасности здравоохранения), опубликованные 107 странами и регионами. Авторами была извлечена информация о заявленных недостатках относительно освещения событий, систем наблюдения за дикой природой и приоритетных болезней. Также были изучены национальные стратегии и планы действий в области биоразнообразия, опубликованные 125 странами, чтобы оценить, включены ли в них мероприятия по эпиднадзору или профилактике заболеваний.

**Результаты:**

Авторы отметили, что частота встречаемости ключевых слов, указывающих на дикую природу, окружающую среду, биоразнообразие и климатические факторы, варьировалась в зависимости от типа отчета и между странами. Было обнаружено, что более половины стран (57,9%, 62/107), опубликовавших отчет, не предоставили ни одного доказательства наличия функциональной программы наблюдения за здоровьем дикой природы. Большинство стран (83,2%, 89/107) указали конкретные недостатки в работе, координировании, сфере охвата или потенциале. Только 8 из 125 стран (6,4%), опубликовавших Национальную стратегию и план действий по сохранению биологического многообразия, сообщили о реальных мероприятиях, связанных со здоровьем дикой природы или зоонозными заболеваниями.

**Вывод:**

В целом, несмотря на их важность для предотвращения пандемии, обсуждения дикой природы и окружающей среды игнорируются в приоритетах и планах обеспечения безопасности здравоохранения. Укрепление потенциала и действий в области охраны здоровья дикой природы должно быть выделено в рамках инициативы ВОЗ «Единое Здоровье» по мониторингу и смягчению известных и новых рисков заболеваний.

## Introduction

Improved health security is crucial for global and national health systems to counter infectious disease epidemics and their wide-scale socioeconomic consequences. The importance of a One Health multisectoral and collaborative approach – one that recognizes the connection between the health of humans, animals and ecosystems – has been acknowledged for years following introduction of the term in the early 2000s.^1^ Although significant advancements in multisectoral coordination have been made over the past decade, the overwhelming focus has been on human and domestic animal health; scant attention has been paid to the risks and impacts of zoonotic diseases at wildlife–human or wildlife–livestock interfaces, or to the role of changing environmental conditions.^2^^,^^3^ The consequences of this neglect have been costly and deadly with thousands of known zoonotic disease outbreaks in recent decades linked to wildlife, for example: human immunodeficiency virus and acquired immunodeficiency syndrome, Lassa fever, Ebola virus disease, highly pathogenic avian influenzas, Nipah virus disease, severe acute respiratory syndrome and coronavirus disease 2019 (COVID-19).^4^^–^^6^

The exclusion of wildlife and environmental issues from global health policies is especially concerning as growing pressures on biodiversity and ecosystems facilitate new or increasing exposure to wildlife, and high mobility through trade and travel enables the rapid spread of pathogens.^6^^–^^8^ A recent analysis based on global change trajectories projected an increase by up to threefold of Ebola virus spillover events and epidemics by 2070.^9^ With an estimated million-plus mammalian viruses still undiscovered, overlooking wildlife health leaves a critical void in health security efforts and a global vulnerability to accidental and intentional sources of biothreats.^10^^,^^11^

Country-level mandates for environmental health are often split across multiple government agencies, with a high potential for fragmentation and gaps, and national funding directed to wildlife health is extremely limited or non-existent.^3^^,^^12^ A review of 18 national action plans on antimicrobial resistance documented the limited integration of environmental considerations, noting that an incomplete application of the One Health approach may miss a key driver and hinder effective control strategies.^13^ This omission for antimicrobial resistance reflects what is perceived as a larger systematic under-representation of the environment sector in health security as a source of unaddressed risks and potential solutions.

National One Health coordination platforms may offer mechanisms to address persistent capacity and implementation needs from all relevant sectors. Doing so will require practical, targeted entry points to integrate environmental expertise and other resources to monitor and manage pathogen spillover risks.^14^ We review relevant national-level assessments and action plans to determine areas of coverage and gaps, and to identify opportunities to integrate the environment sector into global and national health security efforts. We also offer practical recommendations for global and national action to enhance the surveillance of emerging diseases and to improve pandemic prevention and preparedness.

## Methods

There currently exists no capacity assessment tool for national wildlife or environmental services that serves as a parallel to available public health and veterinary services evaluations.^3^^,^^15^ To gauge the extent of wildlife and environmental coverage in zoonotic disease efforts, we therefore reviewed published reports from key processes used to assess national capacity, prioritize national efforts and leverage programmatic funding for health security. Reviewed reports included those published by the World Organisation for Animal Health (OIE) on the Performance of Veterinary Services, and World Health Organization (WHO) Joint External Evaluation missions and follow-on National Action Plans for Health Security (Table 1; available at: http://www.who.int/bulletin/volumes/99/5/20-272690).

**Table 1:** Search strategy and country reports reviewed by type of reporting tool in study of wildlife and environment inclusion in pandemic prevention and preparedness, 2007–2020

Tool	Performance of Veterinary Services	Joint External Evaluation	National Action Plan for Health Security	National Biodiversity Strategy and Action Plan
International body	World Organisation for Animal Health	World Health Organization	World Health Organization	Convention on Biological Diversity
Search terms	English: wild, wildlife, zoonotic and/or zoonoses, environmental, risk factors, risk, drivers, One Health, emerging French: sauvage(s), faune (sauvage), zoonotique, zoonose(s), environnement, risque(s), facteur(s) (de risque, favorisant), une seule santé, émergent(e) Spanish: silvestre, fauna, zoonótico, zoonosis, medio ambiente, ambiente, factor(es) de riesgo, emergente	English: wild animal, wildlife, reservoir, environment, vector, One Health, weather, climate and/or climatological (as “climat” to capture all variations), meteorological (as “meteo”) French: sauvage, faune, réservoir, environnement, environnemental, vecteur, une seule santé, climat, climatique, météo, conditions météorologiques (and relevant variations), temps (in the context of weather and excluding references to time)	English: wild, wildlife, environment, weather, climate and/or climatological (as “climat” to capture all variations), meteorological (as “meteo”), One Health, biodiversity French: sauvage, faune, environnemental, climat, climatique, météo, conditions météorologiques, temps, une seule santé, biodiversité	Wild animal health, wild animal epidemic, wildlife health, wildlife disease, zoonotic, One Health
Additional information compiled	Areas of coordination, diseases under surveillance, gaps	Priority animal or human diseases, best practices, gaps, recommendations for priority actions	None	Specific activities
Countries and territories assessed (listings according to available reports)^a^	Argentina, Australia, Belarus, Belize, Bolivia (Plurinational State of), Botswana, Brazil, Canada, Central African Republic, Chile, Congo, Côte d’Ivoire, Eswatini, Guinea, Guinea-Bissau, Haiti, Iceland, India, Israel, Japan, Kenya, Namibia, New Caledonia, Nigeria, Panama, Paraguay, Rwanda, Seychelles, South Africa, Syrian Arab Republic, Uruguay, Viet Nam	Afghanistan, Albania, Armenia, Australia, Bahrain, Bangladesh, Belgium, Benin, Bhutan, Botswana, Burkina Faso, Burundi, Cambodia, Cameroon, Canada, Central African Republic, Chad, Comoros, Congo, Côte d'Ivoire, Democratic Republic of the Congo, Djibouti, Eritrea, Eswatini, Ethiopia, Finland, Gambia, Ghana, Guinea, Indonesia, Iraq, Japan, Jordan, Kenya, Kuwait, Kyrgyzstan, Lao People's Democratic Republic, Latvia, Lebanon, Lesotho, Liberia, Libya, Lithuania, Madagascar, Malawi, Maldives, Mali, Mauritania, Mauritius, Micronesia (Federated States of), Mongolia, Morocco, Mozambique, Myanmar, Namibia, Niger, Nigeria, North Macedonia, Oman, Pakistan, Philippines, Qatar, Republic of Korea, Republic of Moldova, Rwanda, Saudi Arabia, Senegal, Serbia, Seychelles, Sierra Leone, Singapore, Slovenia, Somalia, South Africa, South Sudan, Sri Lanka, Sudan, Switzerland and Liechtenstein, Thailand, Timor-Leste, Togo, Tunisia, Turkmenistan, Uganda, United Arab Emirates, United Republic of Tanzania, United States, Viet Nam, Zambia, Zanzibar, Zimbabwe	Australia, Benin, Eritrea, Lao People's Democratic Republic,^b^ Liberia, Myanmar, Nigeria, Sierra Leone, Sri Lanka, Uganda, United Republic of Tanzania, United States	Afghanistan, Albania, Angola, Antigua and Barbuda, Armenia, Australia, Austria, Azerbaijan, Bahrain, Bangladesh, Belarus, Belgium, Belize, Bhutan, Bosnia and Herzegovina, Botswana, Brazil, Brunei Darussalam, Cabo Verde, Cambodia, Cameroon, Canada, China, Croatia, Czechia, Democratic People's Republic of Korea, Denmark, Dominica, Egypt, Eritrea, Estonia, Eswatini, Ethiopia, Finland, France, Gambia, Georgia, Germany, Ghana, Greece, Grenada, Guinea-Bissau, Guyana, Hungary, India, Indonesia, Iran (Islamic Republic of), Iraq, Ireland, Italy, Jamaica, Japan, Jordan, Kiribati, Kyrgyzstan, Lao People's Democratic Republic, Lebanon, Liberia, Liechtenstein, Lithuania, Madagascar, Malawi, Malaysia, Maldives, Malta, Mauritius, Micronesia (Federated States of), Mongolia, Montenegro, Mozambique, Myanmar, Namibia, Nauru, Nepal, Netherlands, New Zealand, Nigeria, Niue, North Macedonia, Norway, Pakistan, Palau, Philippines, Poland, Qatar, Republic of Korea, Republic of Moldova, Romania, Russian Federation, Rwanda, Saint Kitts and Nevis, Saint Vincent and the Grenadines, Samoa, San Marino, Sao Tome and Principe, Serbia, Seychelles, Sierra Leone, Singapore, Slovakia, Solomon Islands, Somalia, South Africa, South Sudan, Sri Lanka, Sudan, Suriname, Sweden, Switzerland, Tajikistan, Thailand, Timor-Leste, Trinidad and Tobago, Turkey, Tuvalu, Uganda, Ukraine, United Arab Emirates, United Kingdom, United Republic of Tanzania, Vanuatu, Viet Nam, Yemen, Zambia, Zimbabwe
Total no. countries and/or territories^a^	32	91	12	125^c^
Report years	2007–2019	2016–2019	2017–2019	2010–2020
Accessed on	8 July 2019	13 October 2019	13 October 2019	9 May 2020
Source	https://www.oie.int/solidarity/pvs-evaluations/pvs-evaluation-reports/	https://www.who.int/ihr/procedures/mission-reports/en/	https://extranet.who.int/sph/country	https://www.cbd.int/nbsap/about/latest/

^a^ Country or territory listings as reflected in available reports; grouping may differ by assessment and plan type.

^b^ Lao People's Democratic Republic had a National Plan for Emerging Infectious Diseases, Public Health Emergencies and Health Security implementation mission report posted in lieu of a National Action Plan for Health Security.

^c^ The total number of countries with a National Biodiversity Strategy and Action Plan posted in any language was 169, excluding a report from the European Union.

We conducted a keyword search for terms inclusive of wildlife and environmental risk and monitoring considerations (Table 1). We reviewed documents in their published language (English, French or Spanish) using keyword translations. We interpreted the mention of “animals” to be inherently biased towards domestic animals (pets and livestock, validated by several reports referring to “animals and wildlife”). We therefore screened specifically for “wildlife” and “wild animals”. Given its prominence in the documents and lack of specificity, we did not include the term “zoonotic” in the review of the WHO Joint External Evaluations and National Action Plans for Health Security. We excluded words in standard headings or introductions, as well as non-substantive phrases using keywords in other contexts (e.g. “biosafety environment”). Our review focused on infectious diseases, excluding information on chemical emergencies.

To identify stated weaknesses and evidence of an operational surveillance system for wildlife disease and/or wildlife pathogen screening, we supplemented keyword searches by text review (primarily the chapter on prevention of zoonotic diseases in the Joint External Evaluations for strengths, gaps and recommendations for priority actions). We recorded search terms as “present” or “absent” (available in the data repository).^16^ We did not compare scores from the Joint External Evaluations and Performance of Veterinary Services reports between countries because of recurring updates to evaluation tools and because these indicators were not specifically designed for wildlife or environmental considerations. Furthermore, we did not want to present judgement; our aim is to help to identify weaknesses that can be transformed into opportunities for improving or strengthening health security.

We also extracted information on priority diseases for public or animal health from the Joint External Evaluations (data repository).^16^ Although criteria for priority diseases are not standardized across countries, the evaluations provided an initial indication of the types of diseases considered important in the context of health security.

Despite the fact that the United Nations (UN) Convention on Biological Diversity – the main intergovernmental treaty for biodiversity and ecosystem conservation – has officially recognized the value of a One Health approach, signatory countries are not obliged to consider wildlife health or undertake related activities. To assess the voluntary uptake of wildlife health considerations in conservation planning and commitments, we also reviewed the latest versions of National Biodiversity Strategies and Action Plans submitted under the Convention (if published in English), which serve as the primary mechanism for national implementation (Table 1 and data repository).^16^

All reports mentioned above provide an indication of the primary tools used by external and domestic funders to target investments in health security, animal health, and biodiversity and ecosystem management, and to provide a best estimate of existing efforts and weaknesses.

## Results

### Coverage of topics 

We identified 32 Performance of Veterinary Services reports (published 2007–2019), 91 Joint External Evaluations (2016–2019) and 12 National Action Plans for Health Security (2017–2019) that are publicly available from 107 countries or territories (Table 1 and data repository).^16^ A total of 16 countries (Australia, Botswana, Canada, Central African Republic, Congo, Côte d’Ivoire, Eswatini, Guinea, Japan, Kenya, Namibia, Nigeria, Rwanda, Seychelles, South Africa and Viet Nam) published both Performance of Veterinary Services reports and Joint External Evaluations; 12 countries (Australia, Benin, Eritrea, Lao People's Democratic Republic, Liberia, Myanmar, Nigeria, Sierra Leone, Sri Lanka, Uganda, United Republic of Tanzania and United States of America) published Joint External Evaluations and National Action Plans for Health Security; and two countries (Australia and Nigeria) published all three types of report. 

**Fig. 1:**
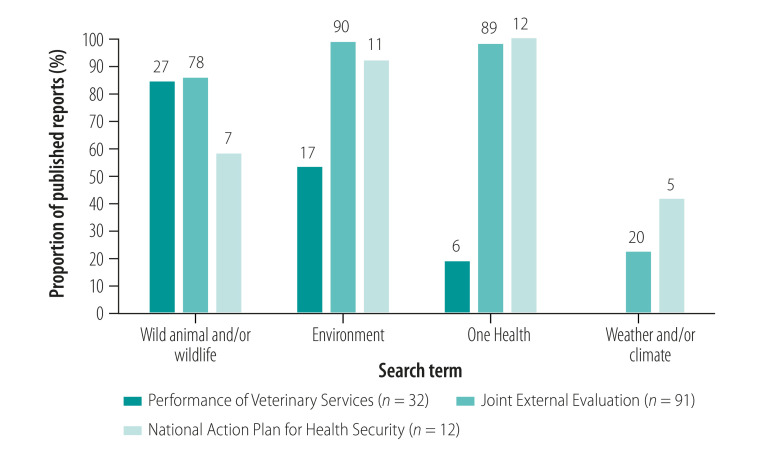
Frequency occurrence of search terms in Performance of Veterinary Services reports, Joint External Evaluations and National Action Plans for Health Security in study of wildlife and environment inclusion in pandemic prevention and preparedness, 2007–2019

Note: The Performance of Veterinary Services reports were not reviewed for the search terms weather and/or climate.

We observed that the occurrence frequency of search terms within reports varied with country and type of report: wild animal and/or wildlife occurred in 58.3% (seven of 12 National Action Plans for Health Security) to 85.7% (78 of 91 Joint External Evaluations); environment occurred in 53.1% (17 of 32 Performance of Veterinary Services reports) to 98.9% (90 of 91 Joint External Evaluations); and One Health occurred in 18.8% (six of 32 Performance of Veterinary Services reports) to 100% (all 12 National Action Plans for Health Security; Fig. 1; data repository).^16^ We noted that the occurrence frequency of climate-related terms varied from 22.0% (20 of 91 Joint External Evaluations) to 41.7% (five of 12 National Action Plans for Health Security; data repository).^16^ More specific search terms used by report type to assess topical coverage ranged from 0% for biodiversity (no mention in any of the National Action Plans for Health Security) to 87.5% for zoonotic and related terms (28 of 32 Performance of Veterinary Services reports) (Table 2).

**Table 2:** Coverage of search terms in specific report types in study of wildlife and environment inclusion in pandemic prevention and preparedness, 2007–2019

Search term	Report type	No. (%) of country reports
Zoonotic and/or zoonoses	Performance of Veterinary Services (*n* = 32)	28 (87.5)
Risk factors	Performance of Veterinary Services (*n* = 32)	4 (12.5)
Vector	Joint External Evaluation (*n* = 91)	76 (83.5)
Reservoir	Joint External Evaluation (*n* = 91)	25 (27.5)
Biodiversity	National Action Plans for Health Security (*n* = 12)	0 (0.0)

### Wildlife surveillance 

From our review of Performance of Veterinary Services reports and Joint External Evaluations published during 2007–2019, of the 107 countries with at least one type of report, 45 (42.1%) provided evidence of a functional wildlife disease surveillance programme or other wildlife-related activities. We noted that the scope varied broadly, and in some cases was limited to selected diseases (e.g. avian influenza surveillance in wild birds). We could not determine the quality or relevance of reported activities, or whether they were sustained over time, highlighting the snapshot nature of assessments and a possible lack of regular communication between sectors. 

Of all assessed countries, 83.2% (89/107) explicitly cited specific gaps or did not include any wildlife coverage. We grouped these cited gaps related to wildlife health into categories most relevant to zoonoses prevention and control (Box 1).

Box 1 **Gaps relevant to wildlife health (as specifically referred to or inferred) in country assessments and plans, and implications, in study of wildlife and environment inclusion in pandemic prevention and preparedness, 2007–2019**

- Poor integration and/or coordination: lack of awareness among sectors; high potential for gaps in mandates, budgets and activities; missed opportunities to synergize
- Not mentioned or insufficient information to assess gaps: wildlife not considered in risk assessments, health security plans or implementation efforts
- Wildlife sector not included in plans, training and/or operations: wildlife and environment considerations omitted; lack of opportunities for wildlife sector to understand relevance to health security; possibly inadequate, inappropriate, inefficient or detrimental disease control measures
- Not operational: no wildlife health and/or disease input to surveillance system; no baseline information; no or low capacity and resources
- Limited capacity (including workforce shortages): inability to perform necessary surveillance and risk reduction activities; insufficient workforce; increased health risks
- Pilot and/or limited scope (disease or geographic scale): surveillance ad hoc and not comprehensive for all relevant species, pathogens and risk interfaces; lack of general surveillance implies poor early-detection capacities; poor understanding and monitoring of disease and/or pathogen occurrence and associated national and transboundary health risks; financing not sustained; capacity and activities may not be transferrable to public sector
- Vulnerability and/or risk targeting only: risk reduction measures not implemented; may not be comprehensive or reflect evolving risks without ongoing monitoring inputs

### Biodiversity 

Our keyword search of the latest English-language National Biodiversity Strategies and Action Plans submitted to the Convention on Biological Diversity revealed that only 8.0% (10/125) of countries included wildlife health and/or zoonotic disease content. When wildlife health content was included, the links between health and biodiversity in the context of disease threats were mainly covered in broad terms (e.g. wildlife–livestock conflicts, wildlife–human interactions, decline of certain species or plant pests). Country-specific wildlife health or programmes and activities related to zoonotic diseases were noted for 6.4% (8/125) of countries (Box 2). One Health was only mentioned in the National Biodiversity Strategy and Action Plan published by Botswana.

Box 2 **List of specific wildlife health activities and programmes included in the latest versions of National Biodiversity Strategies and Action Plans of 125 countries reporting in English, in study of wildlife and environment inclusion in pandemic prevention and preparedness, 2010–2020**

- Brazil: management of the virtual Center for Information on Wildlife Health
- China: monitoring and warning system for pathogenic and epidemic microorganisms with a database, assessment of impacts on biodiversity, and emergency response capabilities
- Islamic Republic of Iran: intended development of a comprehensive plan for emerging environmental issues that includes wildlife diseases
- Liberia: wildlife disease management and surveillance identified as an area of need for long-term expertise training and development
- Republic of Korea: opening and operation of the National Wildlife Health Research Center, establishment of the Second Basic Plan for Wildlife Disease Management, development and implementation of avian influenza countermeasures for wild birds, conducting of surveys and research on wildlife diseases, and strengthening the response and management system
- Rwanda: wildlife health and disease monitoring activities through a nongovernmental organization mentioned under institutional framework for groups involved in biodiversity management
- Uganda: frameworks cited that include prevention and control of diseases presenting a risk to animals and humans
- United Arab Emirates: federal law on zoonotic diseases listed as a relevant policy for the Aichi biodiversity targets

### Priority diseases

Among the diseases of public and/or animal health importance mentioned in the 91 Joint External Evaluations, 40 diseases (or disease categories) were cited by two or more countries (data repository).^16^ Listings ranged from pathogens to specific diseases and syndromes (e.g. viral haemorrhagic fevers). Rabies, brucellosis, anthrax, and avian and/or zoonotic influenza were each listed by at least half of the reporting countries. Countries reported either active surveillance in animals and humans for priority diseases, surveillance of diseases in humans only or that a formal priority disease list had not yet been developed. While transmission dynamics are locally dependent, we noted that the majority of diseases prioritized by countries have environmental determinants that are important for disease management. For example, wild animals play a significant role as reservoirs or maintenance hosts for viral pathogens such as avian influenza viruses (waterfowl),^17^ Marburg and Nipah viruses (certain species of frugivorous bats),^18^^,^^19^ and Lassa and monkeypox viruses (various species of rodents),^20^^,^^21^ reinforcing the potential value of the involvement of the environment and wildlife sectors when designing health security programmes.

## Discussion

Despite the likelihood of devastating impacts from epidemics following a spillover event from wildlife to humans, as has occurred with several emerging diseases in recent decades,^22^^–^^24^ countries are failing to address the environmental components of current health threats. Indeed, our findings indicate that wildlife and environmental considerations remain absent from even the most recent health security capacity assessments and plans. Moreover, for some countries where scientific publications or personal communications report the existence of wildlife health surveillance activities, relevant information was not provided in the official reports. Our findings reinforce the impression that wildlife is not a priority in the context of health security frameworks. Where included, deficits in operations and intersectoral coordination seem to be the rule rather than the exception. Rather than building dedicated systems at country, regional and intergovernmental levels, efforts appear largely ad hoc or driven by external research support. 

It is encouraging that a few countries have set relatively ambitious and specific targets for developing wildlife surveillance frameworks (e.g. Liberia’s National Action Plan for Health Security) and that specific risk interfaces have been identified (e.g. illegal wildlife trade in Viet Nam’s Performance of Veterinary Services report). However, implementation requires dedicated international commitment to support countries in building wildlife health capacity. Even nations with the most developed wildlife health systems acknowledge challenges, gaps and the need for expanded capacity.^25^^,^^26^ Our findings are not meant as a critique of countries, but rather as an opportunity for health security actors to consider new pathways to advance prevention and detection functions by engaging and strengthening other sectors. As National Action Plans for Health Security are branded as multisectoral, all attempts should be made to ensure sufficient inclusion of the environmental sector.

We observed that although countries list several wildlife-associated priority diseases with possibly severe consequences, including the potential to become a pandemic, efforts in health security are generally focused on diseases of domestic animal origin. It is unclear whether this is because of (i) the actual relative disease burden in the country; (ii) the perceived risk or feared impact; or (iii) a bias in assessment and planning processes because of a limited familiarity with monitoring and mitigation measures outside of human health and veterinary services. The lack of attention paid to novel (in particular, wildlife-associated) pathogens, even in recently reported priority disease lists, translates into preparedness deficits from risk reduction activities to diagnostic capabilities. For example, only 9.9% (9/91) of the assessed countries listed coronaviruses or associated diseases as priorities in their Joint External Evaluations, despite prior warnings about the human and animal health threat that they pose.^27^^,^^28^

Several countries prioritize climate- and environment-sensitive diseases (notably echinococcosis, leptospirosis, yellow fever and Rift Valley fever),^29^^–^^32^ but climate is poorly represented in evaluations and plans. Prioritization exercises may benefit from consideration of relevant interfaces and risk factors for emergence, which may be highly country-specific, to guide priority disease selection and appropriate interventions. The anticipated gains may benefit a range of objectives for emerging and endemic diseases, for example: understanding baseline risk; enhancing knowledge of pathogen and host ecology; forecasting and early warning; sentinel detection; and targeting high-risk conditions to reduce the frequency of spillover events.^33^^–^^36^ The evolving threats and uncertainty represented by environmental change will require enhancements in wildlife disease monitoring in both industrialized and non-industrialized settings.^25^^,^^37^

Our review had several limitations. First, for simplicity, we used the terms wildlife health and disease or pathogen surveillance interchangeably. These terms may have nuanced meanings and serve different purposes in practice. For example, wildlife disease surveillance may detect new health threats to animal populations (general, event-based or clinical, and syndromic surveillance), while pathogen surveillance may expand knowledge of pathogens circulating in an area; however, we note that wildlife disease surveillance and pathogen surveillance are complementary, and both are necessary for a comprehensive surveillance programme.^38^ Alternative search terms may have captured a related scope of activities not reflected in our findings. Second, in practice, certain operations may be shared between countries (e.g. surveillance in Liechtenstein and Switzerland). We did not review sources of information on subnational pandemic prevention, as international frameworks (e.g. the International Health Regulations) have emphasized national-level core competencies and, to date, there is no standard subnational approach allowing for comparisons. However, select examples of disease prioritization exercises and provincial programmes have been reported, and will ultimately be critical for expansion and sustainment of efforts.^39^^,^^40^ Third, the static and non-standardized nature of assessments only provides a snapshot of capacity and activities at any point in time; we could therefore not assess progress since the publication of any report. Further, reporting tools were continuously being updated throughout the period covered by the evaluation and planning reports (2007–2020), meaning that measuring progress for any particular country was not possible. Fourth, we acknowledge possible reporting gaps (> 100 countries completed Performance of Veterinary Services reports, but only 32 elected to make their reports public). Furthermore, we did not perform a systematic assessment of all gaps present, but rather inferred the main gaps based on those recognized and self-reported in reports; our interpretations may therefore be subjective. However, our findings are consistent with the well-recognized and chronic gaps in surveillance of wildlife diseases previously reported by others,^26^^,^^41^ and highlight the need for further assessment and benchmarking of wildlife health systems to reduce pandemic risk.

Our study benefited from several strengths. First, rather than relying on complex modelling tools, the evidence in the self-reported assessments was straightforward to extract without the introduction of errors or possibility of misinterpretation. Second, our inclusion of evaluations and plans from multiple sectors – human health, animal health and the environment – is consistent with the One Health approach needed to examine this topic. 

Overall, the necessity of multisectoral collaboration for health security is increasingly recognized. The UN General Assembly resolution A/74/306, adopted in September 2020, calls for a climate- and environment-sensitive approach to building back better in COVID-19 recovery efforts, including the protection of wildlife.^42^ However, in its 2020 Annual Report, the Global Preparedness Monitoring Board issued a call for “robust global governance”, including predicting and detecting pathogen emergence via a One Health approach, broadly mentioning human and animal health but lacking any call to action for the environment sector to specifically engage in preparedness initiatives.^43^ This absence, alongside emphasis on preparedness for health emergencies rather than risk reduction, demonstrates that dedicated effort is required to correct for the continued omission of the wildlife and environment sectors in recovery from the COVID-19 pandemic. Accordingly, a report issued by the Intergovernmental Science-Policy Platform on Biodiversity and Ecosystem Services has noted the potential value of a shift towards pandemic prevention that explicitly integrates biodiversity and health science to inform decision-making.^8^

A key challenge is that an institutional mandate for wildlife health is not captured by any specific intergovernmental agency. Without formal responsibility for wildlife health, there are significant gaps in the implementation of surveillance programmes spanning risk assessment, reporting, investigation and management decisions. A responsible authority and budget source for all wildlife and environmental health functions may not be readily identified, given the typical scope of natural resource management (e.g. biodiversity and ecosystem monitoring may be in place, but not disease/pathogen surveillance) and limited inclusion of wild animals in veterinary services.^12^ A review of mandates is needed to assign responsibility and develop plans for short-term pragmatic stopgaps and long-term capacity and workforce strengthening.

As a first action, existing health security programmes should be reviewed for targeted entry points for the environment sector. At a global level, a dedicated intergovernmental environment partner is needed to ensure representation in relevant decisions, guidance and programmes. Expanding on prior intergovernmental coordination,^44^ the launch of a high-level One Health expert group has been proposed for 2021 by WHO, OIE, UN Food and Agriculture Organization, and UN Environment Programme.^45^ Success will require sustained efforts and resourcing, equitable representation and, ideally, alignment with the sustainable development goals for broader benefits.

Evidence syntheses and guidance resources have been produced through the Convention on Biological Diversity–WHO Joint Work Programme on Biodiversity and Health, with the Convention also adopting guidance on integrating biodiversity considerations into a One Health approach.^46^^,^^47^ Efforts are needed to systematically translate agreements and knowledge products into country-level planning and implementation. One possible path is via add-on funding of existing bilateral or multilateral health security projects at national or regional levels, such as the World Bank Regional Disease Surveillance System Enhancement programme that supports human and animal health systems and multisectoral coordination mechanisms in 16 central and west African countries.^48^

Several nominal changes can help ensure the visibility of wildlife and of broader considerations for environmental risk mitigation in health security. We encourage public health and animal health sectors to examine existing tools and programmes (e.g. Performance of Veterinary Services reports and Joint External Evaluations) in the immediate term, and to continue to take steps to support their line ministries in identifying areas of value for collaboration with the environment sector. For example, the OIE has developed a framework to enhance veterinary service capacities in managing risks from emerging diseases while protecting wildlife.^49^ Assessment and operational tools have evolved in important ways since their first iterations to encompass greater scope, signalling that future editions may facilitate meaningful intersectoral collaboration. Future versions can expand on competencies for risk reduction, particularly those related to disease emergence. Specifically, greater attention must be paid to wildlife and environmental change as the major source of emerging zoonoses.^4^ Similarly, involving both public health and animal health authorities in the design and implementation of National Biodiversity Strategies and Action Plans may enhance the health security value of biodiversity-focused activities. We provide a set of initial ideas for developing this field at different levels (Box 3). Support to countries in systematic planning and pragmatic implementation could be catalysed through a multisectoral health security convening and priority-setting body, such as the Global Health Security Agenda.

Box 3 **Recommendations to strengthen wildlife health capacity arising from study of wildlife and environment inclusion in pandemic prevention and preparedness, 2020**

**Global infrastructure**

- Partnerships: empower environment entities to contribute to intergovernmental One Health and health security initiatives to ensure equal and systematic inclusion
- Assessment and planning tools: (i) include a dedicated space for a wildlife health expert in Joint External Evaluation and Performance of Veterinary Services assessment teams, and involve zoonotic disease experts in National Biodiversity Strategies and Action Plans; (ii) add framing or questions specific to wildlife, and relevant risk monitoring and mitigation of environmentally sensitive wildlife or vector-borne diseases; and (iii) develop an assessment tool to target wildlife and ecosystem management capacity to supplement public health and veterinary services assessments
- Resource mobilization: dedicate funding to wildlife health capacity development, or target a portion of One Health funds to be directed to implementation activities
- Investigation and reporting: implement systems for (i) immediate notification of wildlife mass morbidity or mortality events (annual reporting of selected wildlife disease events to the OIE is currently voluntary via the World Animal Health Information System - Wild Interface); and (ii) investigation of wildlife disease events (parallel to the Global Outbreak Alert and Response Network in human health events)
- Training: (i) offer field-based epidemiology training programmes for wildlife veterinarians; and (ii) report on number of qualified wildlife veterinarians and/or wildlife health professionals by country (parallel or subset of veterinarians and para-veterinarians reporting to the OIE)
- Implementation: develop guidance for accessing and interpreting wildlife health data to assess threat to public health, domestic animal health, and biodiversity and ecosystems

**National and subnational**

- Planning: develop wildlife health sector (institutional mandates, training, resourcing and workforce development)
- Reporting: establish mechanism(s) for centralized reporting of wildlife health and/or disease research to a national entity
- Risk assessment and monitoring: (i) set up arrangements with laboratories for testing of wildlife samples (and implement appropriate export and import agreements if international); (ii) perform risk profiling and assessment of major wildlife–domestic animal and wildlife–human interfaces (e.g. bushmeat markets) to identify high-risk transmission interfaces; (iii) perform risk profiling and assessment for diseases in native and introduced wildlife species to inform conservation planning, livestock biosecurity and zoonotic disease prioritization; (iv) require consultation of government wildlife entity(ies) or expert scientists in case of human or domestic animal disease connected to environmental resources; and (v) integrate wildlife and other environment information into a surveillance system leveraging local stakeholders (e.g. park rangers, community eco-monitors and hunters)

OIE: World Organisation for Animal Health.

As evidenced by the COVID-19 pandemic, there are unacceptable risks in neglecting the wildlife and environmental drivers of pathogen spillover. Wildlife health surveillance is paramount to the success of the One Health movement in preventing, detecting and mitigating known and novel zoonotic disease risks. We appeal to countries and multisectoral panels to urgently acknowledge and remediate these gaps in global and national health security priorities and efforts.
